# Relationship between Specific Field-Based Physical Fitness Test Results and Selected Health Biomarkers in College-Aged Males: A Cross-Sectional Study

**DOI:** 10.3390/ijerph192114498

**Published:** 2022-11-04

**Authors:** Pablo Prieto-González

**Affiliations:** Health and Physical Education Department, Prince Sultan University, Riyadh 11586, Saudi Arabia; pprieto@psu.edu.sa; Tel.: +966-114-948-661; Fax: +966-11-454-8317

**Keywords:** fitness test, physical condition, anthropometric, health biomarkers

## Abstract

Objective: This study aimed to verify the association between specific field-based physical fitness test results and selected health biomarkers in college-aged males. Method: A total of 390 males participated in this research. The association between fitness test scores and anthropometric and health variables were examined. The fitness tests conducted were: Sit-and-reach test (S&R), standing long jump test (SLJ), Shuttle run test (SHR), and 20 m Multistage Fitness Test (BT) to estimate the maximum oxygen uptake (VO2max). The anthropometric and health variables assessed were: Weight (WE), height (HE), body mass index (BMI), body fat percentage (FAT), lean body mass (LBM), abdominal Girth (AG), waist-to-hip ratio (WHR), systolic blood pressure (SBP), diastolic blood pressure (DBP), oxygen saturation (SPO2), average blood pressure (A-BP), double product (DP), and fasting blood glucose (GLU). Results: S&R presented a weak significant correlation with SLJ, VO2max, and AG. SLJ maintained weak to moderate significant correlations with S&R, SHR, VO2max, HE, WE, LBM, WHR, BMI, FAT, AG, SBP, DBP, A-BP, DP, and GLU. SHR presented weak to moderate significant correlations with SLJ, VO2max, WE, BMI, AG, FAT, HE, SBP, DP, and GLU. VO2max maintained weak to moderate correlations with S&R, SLJ, SHR, WE, BMI, FAT, LBM, AG, and DP. Weak to moderate correlations were found between anthropometric and health variables, whereas the anthropometric variables presented significant correlations with each other, ranging from weak to very strong. Fitness test results presented weak to moderate correlations among themselves. Conclusion: SLJ and SHR present weak to moderate validity to predict the selected anthropometric markers and weak to predict the selected health indicators except for SPO2. VO2max has only weak validity to predict the selected anthropometric markers, whereas S&R is not valid to predict the selected health or anthropometric markers. Anthropometric measurements have weak validity in predicting the selected health markers. BMI and AG are valid, simple, and economical measurements to assess body fat. A positive interaction between the results obtained in the field-based fitness tests conducted was observed.

## 1. Introduction

Health is defined as a state of complete physical, mental and social well-being and not merely the absence of disease or infirmity” [1]. To promote it, the World Health Organization, public authorities, health professionals, and Physical Education teachers foster physical activity to improve citizens’ physical fitness and health conditions [2,3]. Then, to assess the improvements attained through the practice of physical activity (particularly in physical health), Physical Education teachers and sports coaches regularly use fitness tests to assess subjects’ physical fitness. However, the absence of valid measurement instruments is one of the main challenges in the field of physical activity and sports sciences [4,5,6]. Therefore, it is necessary to have simple, inexpensive, and non-invasive tests to assess the health-related physical fitness of athlete and non-athlete populations [7].

Physical fitness can be objectively assessed using laboratory and field tests. Laboratory tests offer the advantage of being conducted under highly controlled conditions, provide a wealth of data, and are accurate [7,8,9]. However, they also present some disadvantages, such as economic cost, being time-consuming, and requiring qualified technicians for their implementation [7,8,9]. Therefore, using them in school or recreational contexts, or in epidemiological studies is not feasible. Thus, field tests represent an excellent alternative to laboratory tests due to their easy execution, low economic cost, absence of sophisticated technical equipment, and low time required to conduct them. They are also useful to evaluate simultaneously many participants [7,8,9].

Ruiz et al. (2011) [10] consider that using fitness tests, assessing the physical condition and the levels of physical activity at the population level must be a priority for the health authorities. They argue that physical inactivity and poor physical fitness are associated with a higher probability of suffering from certain non-communicable diseases (i.e., cardiovascular diseases, diabetes, cancer) and have a negative effect on the risk factors of these diseases (blood pressure, high levels fasting blood glucose and obesity), which impacts negatively on the health of the entire population. In the same vein, the Physical Education Curriculum of Secondary Schools in countries such as Spain and the United Kingdom underlines the need to improve physical fitness to attain adaptations that result in the enhanced health status of the students [11,12].

However, whether physical fitness test results are associated with selected health indicators is questionable, considering health indicators as a way of measuring specified health characteristics in a given population [13,14]. On the one hand, some studies have examined the interactions between physical activity, physical fitness, and health in children and adolescents to verify whether physical fitness constitutes an important and valid health biomarker [8,10,15,16]. Based on the results of these studies, it was found that cardiovascular fitness maintains a directly proportional relationship with resting heart rate and with a better cardiovascular profile, while it maintains an inversely proportional relationship with abdominal adiposity, body fat percentage, body mass index, body weight, and waist circumference [10,15,16]. It was also verified that muscular fitness positively affects skeletal health, cardiovascular profile, and overall adiposity [10,15,16]. Some studies have also ascertained that speed/agility is associated with better skeletal health [10,16]. On the other hand, according to Ruiz et al. (2009) [16], whether physical fitness is an important marker of health in childhood and adolescence is still under debate. In fact, Suni et al. (1998) [17] found that leg strength and leg flexibility in men; and leg power, trunk, and leg flexibility in women were not associated with health outcomes. In addition to the absence of correlation between physical fitness test results and health parameters found by these authors, Martínez-Vizcaíno and Sánchez-López (2008) [18] argue that the existence of conflicting results and the great variability of physical fitness measurements in children and adolescents could be related to the multitude of methods used in their assessment.

In this context, additional studies are required to analyze the association between the status of different fitness components (i.e., endurance, strength, flexibility, and speed/agility) and the health condition, assessing a comprehensive number of health and anthropometric parameters. This information will help clarify whether physical fitness tests constitute a valid and low-cost alternative to laboratory tests in contexts where many individuals are tested simultaneously in small spaces, with limited time, and where advanced medical equipment and personnel trained in exercise physiology are unavailable. Therefore, the objective of the present study was to verify the existing association between specific field-based physical fitness test results and selected health biomarkers in college-aged males.

## 2. Materials and Methods

### 2.1. Type of Study

A cross-sectional study was conducted following the STROBE guidelines. It was carried out in accordance with the principles outlined in the Helsinki Declaration. It will also be approved by the Institutional Review Board of the Bioethics Committee at Prince Sultan University.

### 2.2. Participants

Three hundred ninety college-age males voluntarily participated in the present study. They were properly informed about the objectives, benefits, and risks of participating in this research. To be included, they signed an informed consent form indicating their willingness to participate. Study participants were informed that they could withdraw from the study without penalty at any time. The subjects’ anonymity was guaranteed, and the recorded information was exclusively used for scientific purposes. The study participants did not receive any compensation for participating in the current research. Inclusion criteria were: (a) Male; (b) Age 17–21 years; (c) Resident in Riyadh; (d) Neither suffer from genetic, endocrine, mental, or degenerative diseases, nor from traumatic injuries incompatible with the performance of the physical fitness tests conducted; (e) Do not practice structured or planned physical exercise; (f) BMI ≤ 30, (since obesity is also considered a chronic multifactorial disease) [19]. The study was carried out in Riyadh between 1 December 2021 and 31 January 2022. The sample selection was probabilistic. Thus, a stratified sample of the 15 districts that make up the city was used. The subjects were invited to participate in the present study through the Riyadh University forums available on social networks (WhatsApp and Facebook Messenger). The sample size was determined using the following formula [20]:

n = z^2^ * × p × q × N/e^2^ (N − 1) + z^2^ × p × q

where n = sample size; z = confidence level; p = probability of success; q = probability of failure; N = population size; e = confidence interval. The confidence level was set at 95%, and the confidence interval at 5%. As for the probability of success, 50% was chosen since it is the most conservative option to maximize the sample size. After that, it was ascertained that the number of individuals required to have a representative sample of the population studied was 384.

### 2.3. Assessments

All measurements (anthropometric and health parameters and physical fitness tests) were performed by the same researcher to achieve greater reliability and accuracy.

#### 2.3.1. Anthropometric and Health Variables

Anthropometric measurements were made following the ISAK standards. An indoor facility with a temperature between 20 and 22 degrees was used. The assessments were conducted between 9.00 am and 11.00 am on an empty stomach and were taken in the right hemisphere of the body. Except for the blood fasting glucose, all parameters were assessed three times, and the value finally registered was the median of the three measurements. All devices used were previously calibrated with a sample of 25 subjects. Body mass (BM), height (HE), and body mass index (BMI) were assessed with a Seca digital column scale (Hamburg, Germany). BM was recorded to the nearest 0.1 kg, and HE to the nearest 0.1 cm. The measurements were taken with the subjects barefoot and in light clothing. Body fat percentage (FAT) was estimated using the following formula [21]:

FAT = ((Σ of abdominal, supraspinal, subscapular, triceps, quadriceps, and calf skinfolds) × 0.143) + 4.56

The skin folds were measured with an FG1056 Harpenden skinfold caliper (Sussex, UK). Lean mass (LEAN) was estimated with the following formula:

LT = total weight (kg) − fat mass (kg)

Abdominal girth (AG) and waist-to-hip ratio (WHR) were measured with a Seca 203 Ergonomic Circumference Measuring Tape (Hamburg, Germany). Afterward, WHR was estimated with the following formula:

WHR = waist circumference (cm)/hip circumference (cm)

Systolic and diastolic blood pressure (SBP and DBP) were assessed with the study participants relaxed and seated utilizing an Omron Upper Arm Gold Blood Pressure Monitor (Kyoto, Japan). Three measurements were taken at intervals of 1 min [22]. The double product (DP) was calculated using the following formula:

Double product = systolic blood pressure × heart rate

Oxygen saturation (SPO2) was measured on the index finger, with the patient also seated and relaxed using a BPL Smart Oxy Lite Pulse Oximeter (Arakere, Bangalore, India). Similarly, average blood pressure (A-BP) was calculated with the following formula [23]:

A-BP = Diastolic pressure + (1/3 × Pulse Pressure)

Fasting blood glucose (GLU) was measured with a Benecheck Meter Kit (New Taipei City, 242 Taiwan).

#### 2.3.2. Fitness Tests

A reduced version of the Eurofit battery was applied to evaluate the physical fitness of the study participants. The tests selected were: Sit-and-reach test (S&R), standing long jump (SLJ), 5 × 10 shuttle run test (SHR), multi-stage fitness test or beep tests (BT). Before performing the tests, the subjects underwent a 15 min warm-up. It consisted of the following three phases: (1) Activation: Five minutes of progressive running. (2) Join mobility: Participants mobilized each join 10 times following a head-to-toe order. (3) Specific phase: Subjects performed 2 × 10 jump squats, and two sprints of 20 m distance. Similarly, study participants were asked not to perform strenuous physical exercise 48 h prior to performing the physical test battery. They were also instructed not to consume caffeine 24 h before testing. Similarly, subjects were advised to be properly hydrated. The fitness tests were performed on two different days. On day 1, S&R and BT, and on day 2, 48 h later, SLJ and SHR.

S&R: This test was used to evaluate flexibility, particularly hamstring extensibility [24]. The device used was a Baseline Sit and Reach Trunk Flexibility Assessment Testing Box (Sacramento, CA, USA). The test started with the participant seated on the floor with his knees close together and fully extended. Next, the sole of his feet was placed flat against the sit-and-reach assessment testing box. Next, he placed his hands on top of the box, and in a slow, steady movement, he reached forward as long as possible, sliding his hands by bending his trunk and keeping his knees straight. This position was held for at least 5 s. At this point, the researcher marked the distance at which the participant’s fingers reached from his toes. Results were rounded to the nearest 0.5 cm to register the mark obtained. Positive results were obtained when the participants reached beyond their toes, and negative when they could not [25]. Two attempts were allowed, and the best one was registered.

SLJ: It was used to assess the lower body’s explosive power. The material used was an Atreq standing long jump mat (North Yorkshire, UK). The participant stood behind the starting line on the mat, with his feet slightly apart. After bending his trunk and legs and balancing with his arms, he was instructed to jump as far forward as possible. The distance between the take-off line of the mat and the rear back of his shoes was measured. Results were rounded to the nearest 0.5 cm to register the mark obtained. The jump was considered invalid if the participant: (a) jumped again after landing; (b) stepped on the white line during the take-off; (c) did not jump or land on both feet simultaneously (Reid et al. 2017) [26]. Two attempts were allowed, and only the best was recorded.

SHR: This test was used to assess speed and agility. It was performed in a non-slip indoor gymnasium. The subject had to run back and forth over 5 m, for a total of 50 m, between two lines placed 5 m apart and marked with cones, with an outside margin of 2 m. The test began with one foot of the study participant on the starting line. After the starting signal, the subject ran to the opposite line and then back to the starting line. This was repeated five times without stopping (covering a total of 50m). At each marker, both feet had to cross the line completely. The result was the time taken to complete the 50 m [27,28]. The time was recorded using a Casio^®^ HS-80 TW-1EF timer, Shibuya, Tokyo, Japan. Each subject had two attempts—with a rest interval of three minutes—and only the best result was registered.

BT: It was used to estimate cardiorespiratory endurance and maximal oxygen uptake (VO2max). It is a back-and-forth incremental, continuous, maximal-to-fatigue running test. It was performed on a flat, non-slip surface of 27 m wide. Study participants ran back and forth between two lines set 20 m apart at a pace dictated by audio. After one minute, a sound indicated an increase in speed, and the beeps were closed together. The initial speed was 8.5 km h^−1^, and it was increased by 0.5 km h^−1^ every minute. Subjects were required to step behind the 20 m line when the tones were emitted. When the line was reached before the sign sounded, they were instructed to wait until hearing the tone before continuing. Conversely, when the line was not reached before the tone sounded, study participants were given one warning and had to continue running to the line and try to catch up with the pace within two more tones. Subjects received the first warning the first time they failed to reach the line. The test ended when the participants received the second warning or stopped due to fatigue. Only one attempt was allowed. The athlete’s score was the number of shuttles completed before finishing the test [29,30]. Subsequently, VO2max was calculated using the following formula [30]:

VO2max = 0.0276x + 27.504

### 2.4. Statistical Analysis

The results are shown using the format mean SD (standard deviation). The normality of the data was assessed using the Kolmogorov–Smirnov, whereas homoscedasticity was confirmed using Levene’s tests. The bivariate Pearson correlation analysis was used to verify the association between continuous variables. Pearson correlation coefficients were interpreted as follows: r = 0 null correlation; 0.01 ≤ r ≤ 0.09 very weak; 0.10 ≤ r ≤ 0.29 weak; 0.30 ≤ r ≤ 0.49 moderate; 0.50 ≤ r ≤ 0.69 strong; and r ≥ 0.70 very strong [31]. To explain the nature of the relationship between certain variables, a multiple linear regression analysis using the stepwise method was performed. For this purpose, the results obtained in the fitness tests were used as dependent variables and all anthropometric and health variables as independent variables. The Durbin-Watson statistical test was performed in parallel to this test to verify the possible existence of spurious relationships between variables. A binary logistic regression test was also performed to verify whether the results obtained in the selected fitness tests could predict specific health indicators. For this purpose, the variables BMI, FAT, AG, WHR, SBP, DBP, SPO2, and GLU were used as dependent variables, and the results attained in the fitness tests included in this study (S&R, SLJ, SHR, BT) were used as predictor variables. Likewise, since the dependent variables used in the logistic regression model conducted must be binary, the values that may be considered as “healthy or acceptable” and “unhealthy or of increased risk” were established according to the current scientific evidence (see Table 1) [32,33,34,35,36,37].

After performing the logistic regression analysis, the Nagelkerke R^2^ values obtained were interpreted as follows: poor (0–0.1), modest (0.1–0.3), moderate (0.3–0.5) and strong (>0.5) model accuracy [38]. The level of significance established was set at *p* < 0.05. The statistical analysis was performed using the program IBM SPSS V.26^®^ computing (IBM Corp., Armonk, NY, USA).

## 3. Results

The characteristics of study participants and the results they obtained in the anthropometric, health, and fitness assessments are shown in Table 2. The correlation matrix is shown in Table 3.

**Figure 1:**
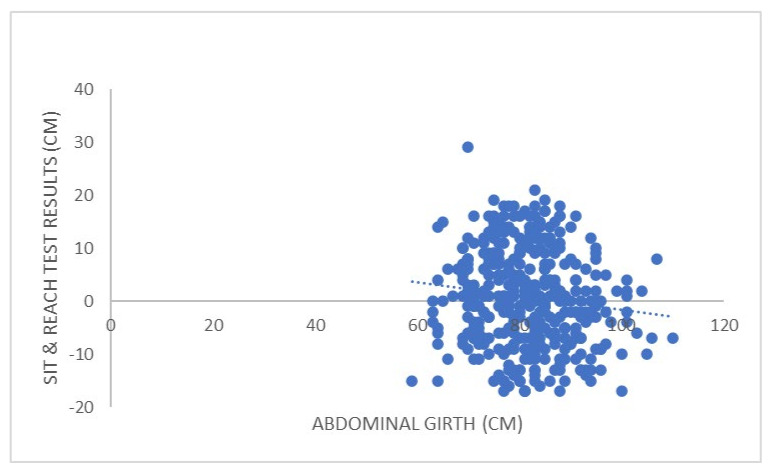
Correlation between the results obtained by the study participants in the sit-and-reach test and abdominal girth (the only parameter with which maintained a statistically significant correlation).

**Figure 2:**
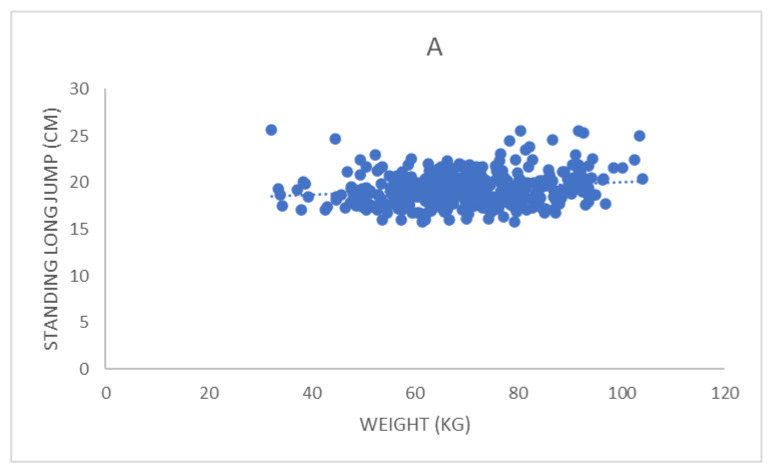

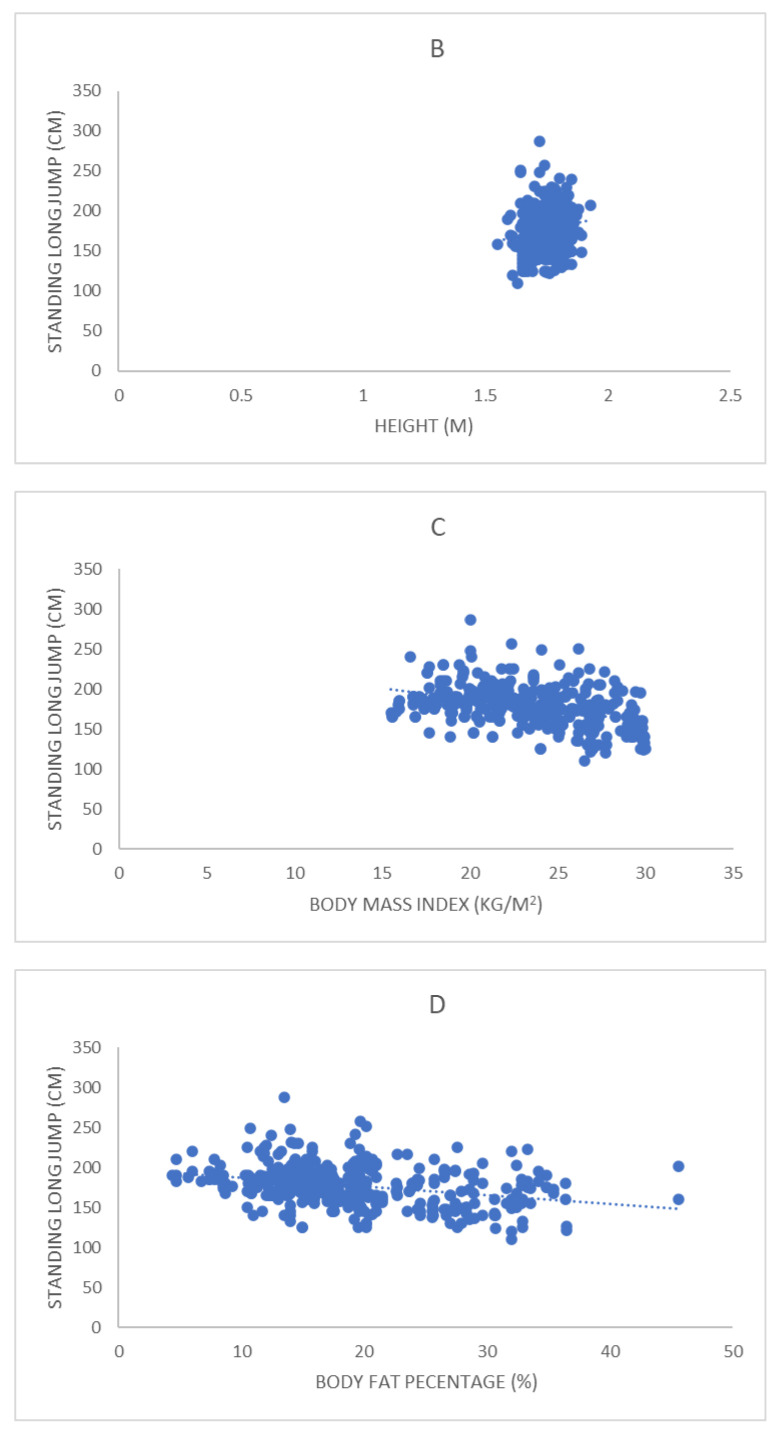

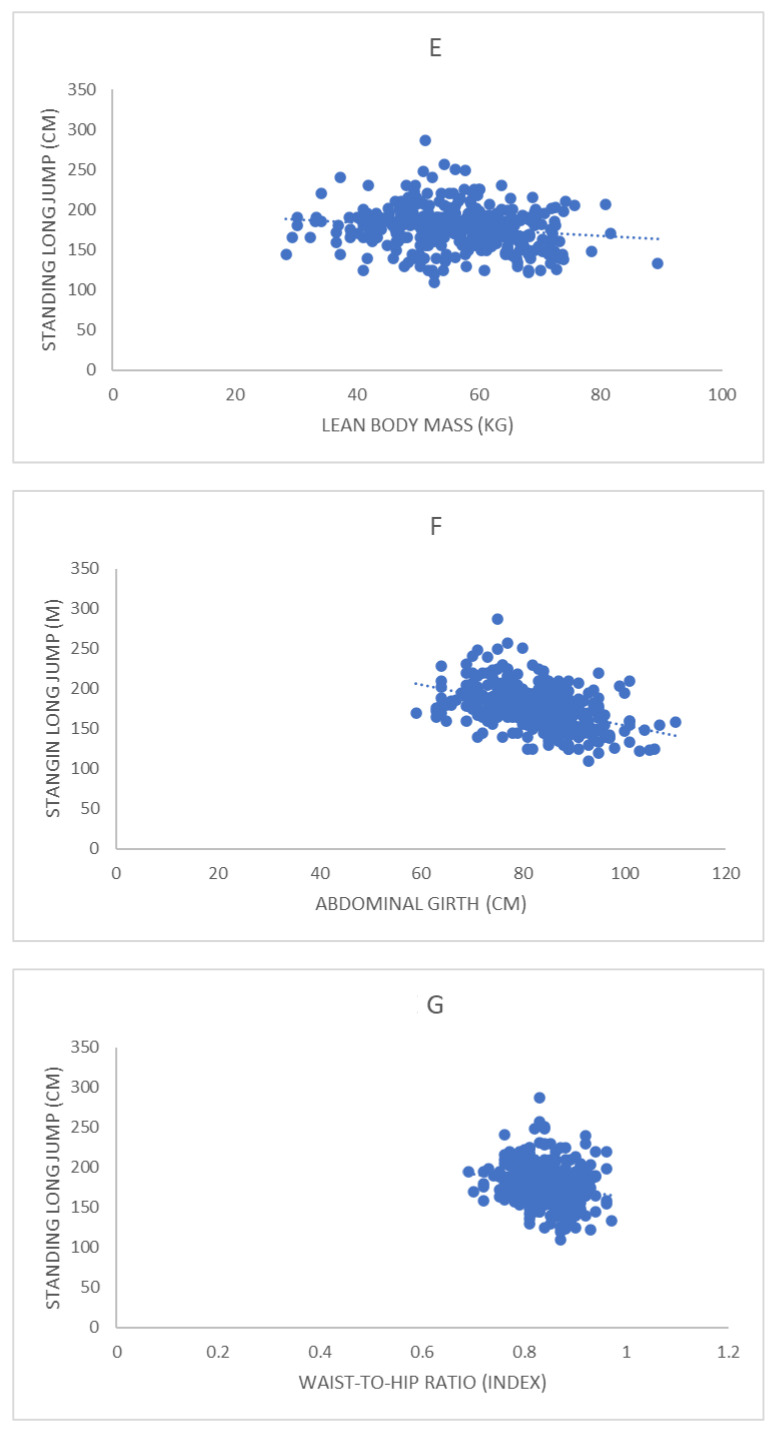

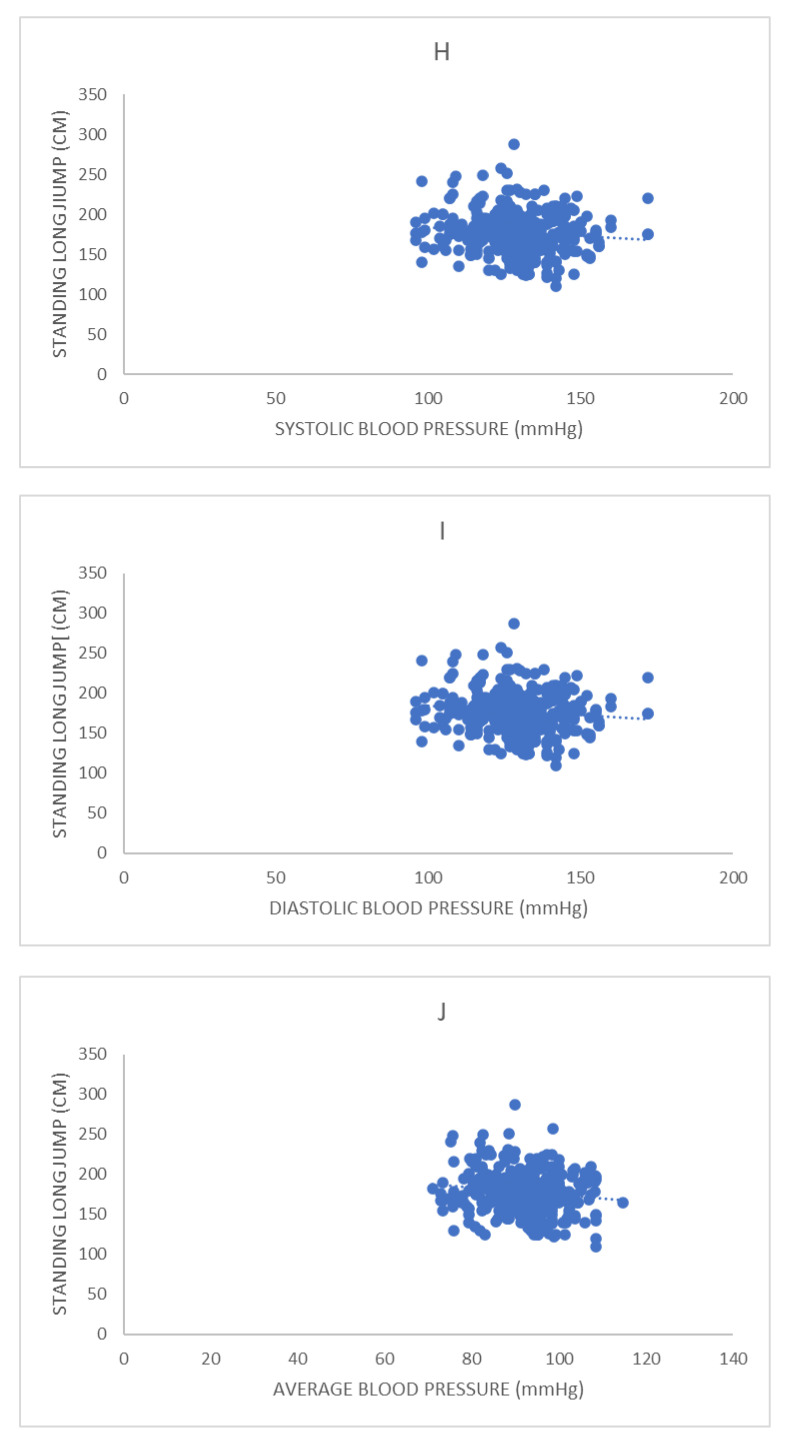

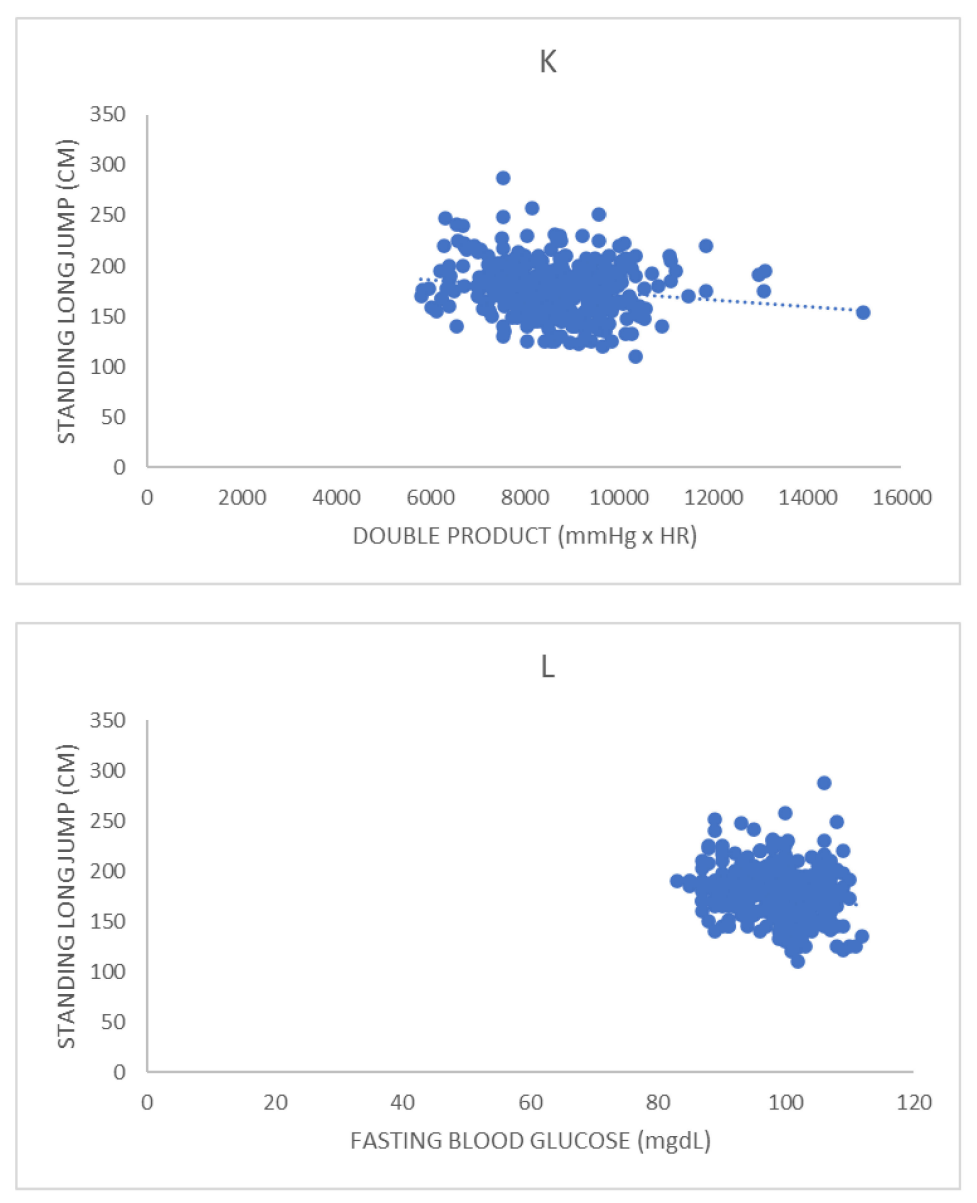
Correlation between the results obtained by the study participants in the standing long jump test and the anthropometric and health variables with which it maintained a significant correlation: (**A**) weight; (**B**) height; (**C**) body mass index; (**D**) body fat percentage; (**E**) lean body mass; (**F**) abdominal girth; (**G**) waist-to-hip ratio; (**H**) systolic blood pressure; (**I**) diastolic blood pressure; (**J**) average blood pressure; (**K**) double product; (**L**) fasting blood glucose.

**Figure 3:**
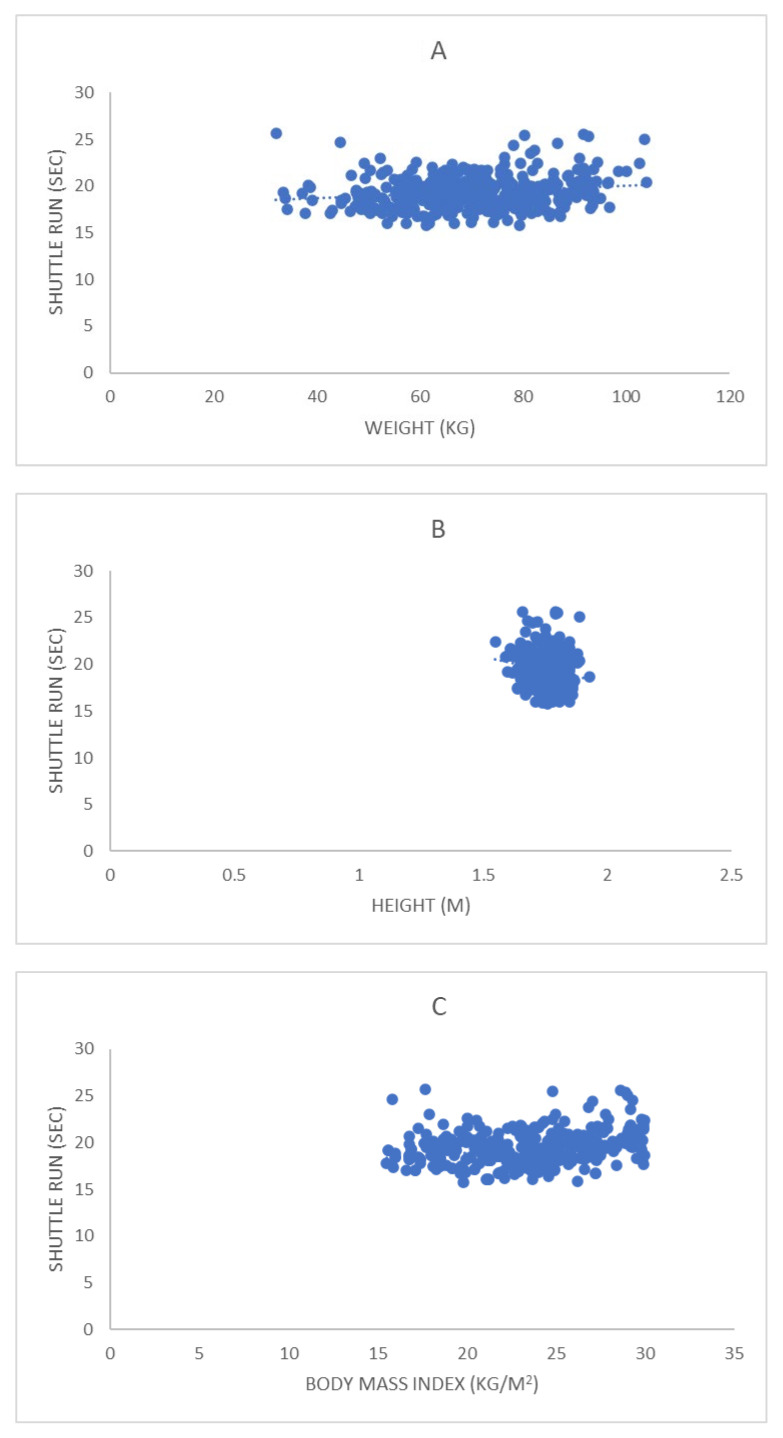

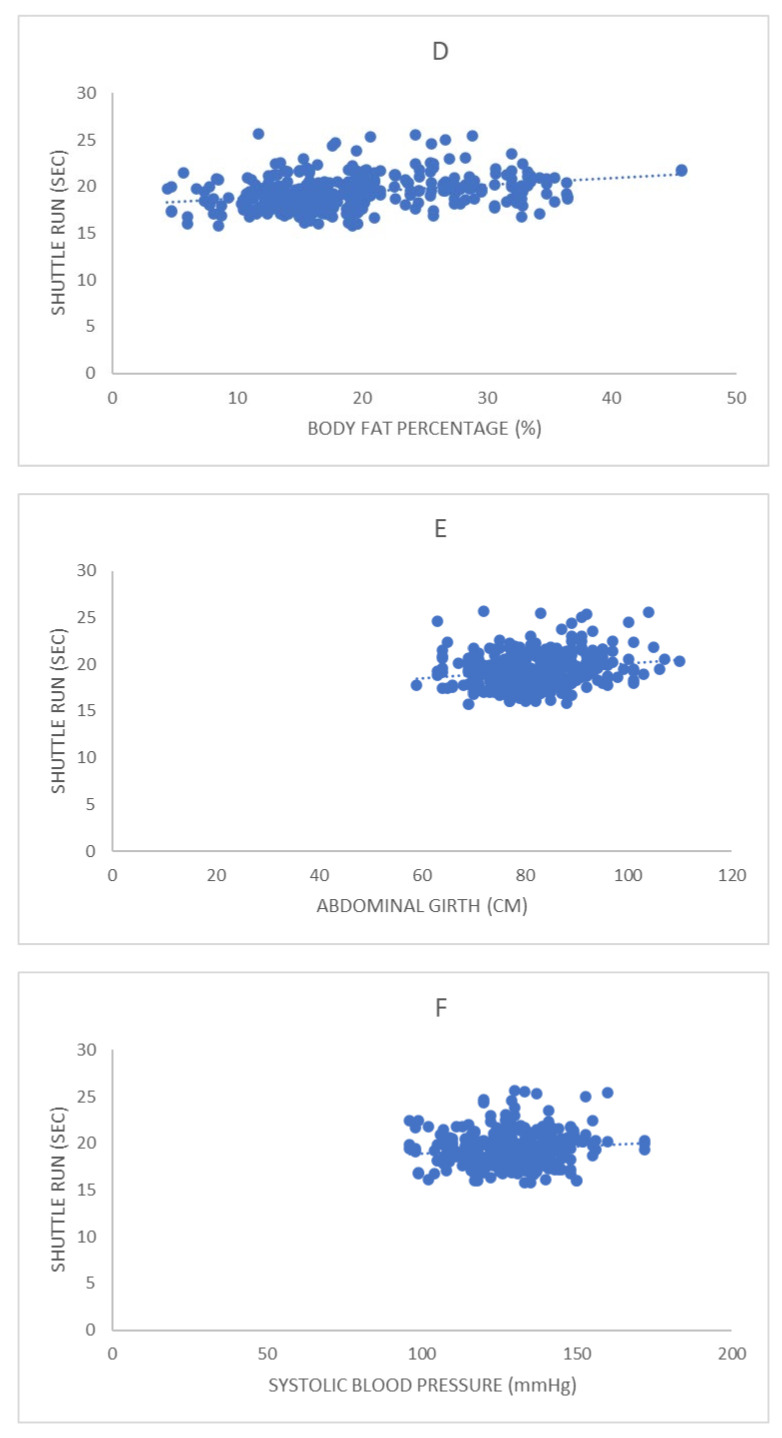

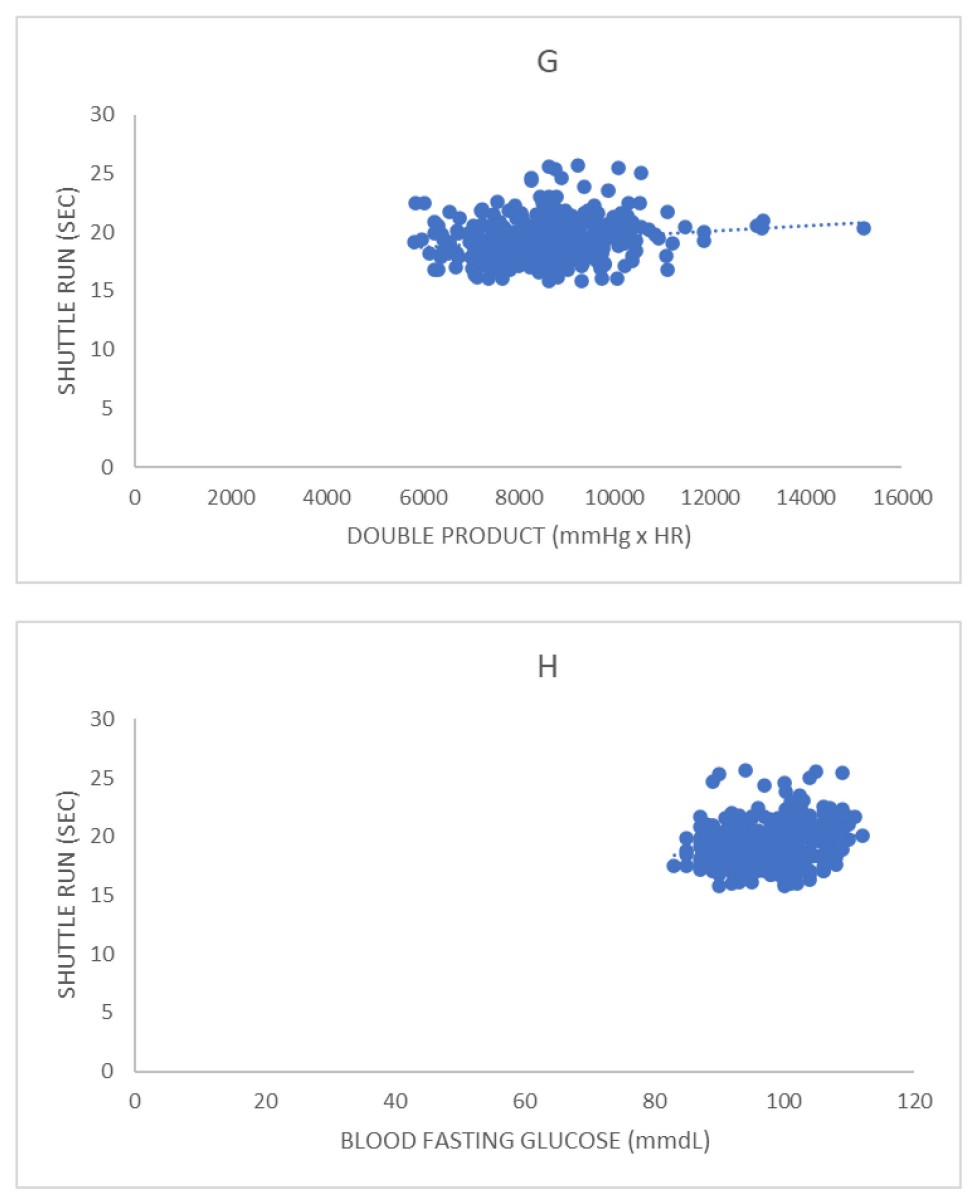
Correlation between the results obtained by the study participants in the shuttle run test and the anthropometric and health variables with which it maintained a significant correlation: (**A**) weight; (**B**) height; (**C**) body mass index; (**D**) body fat percentage; (**E**) abdominal girth; (**F**) systolic blood pressure; (**G**) double product; (**H**) fasting blood glucose.

**Figure 4:**
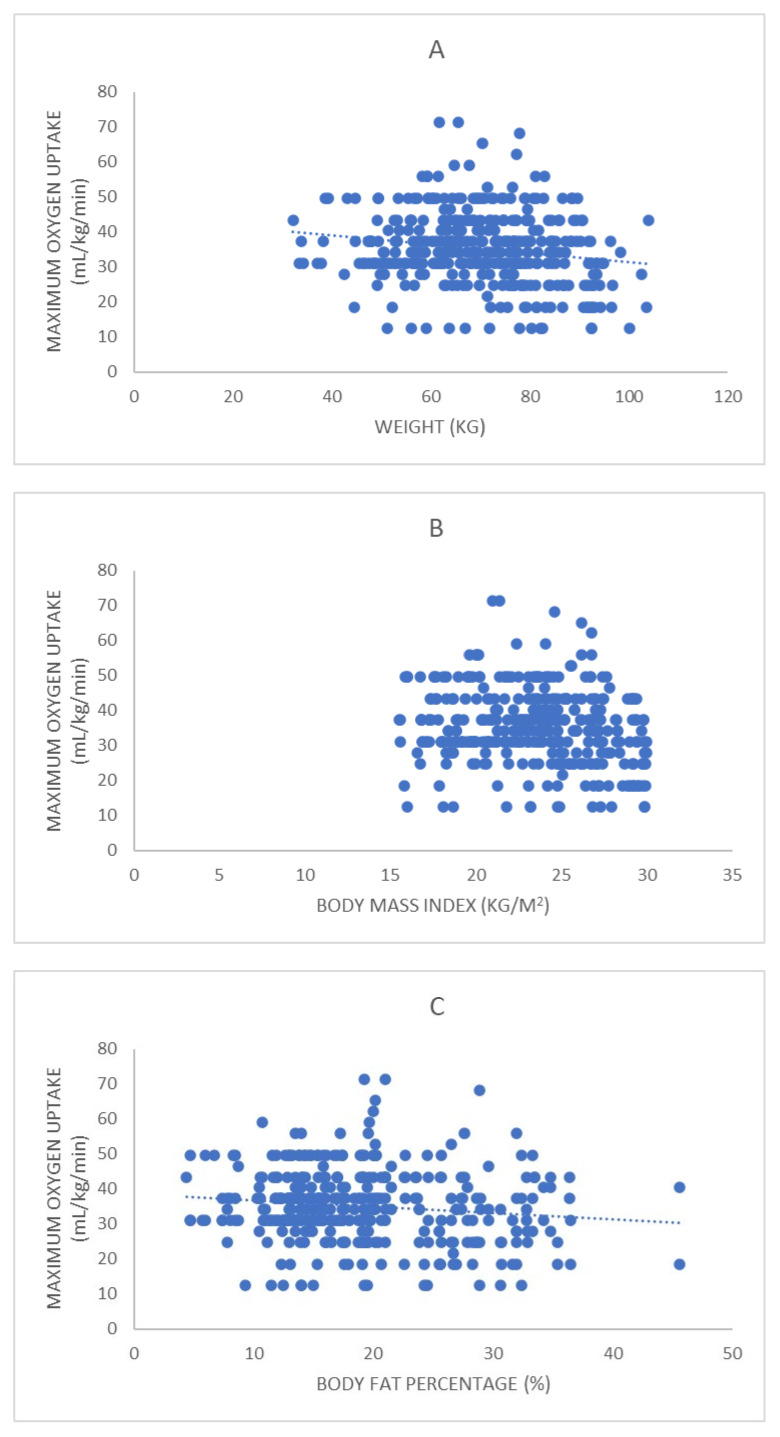

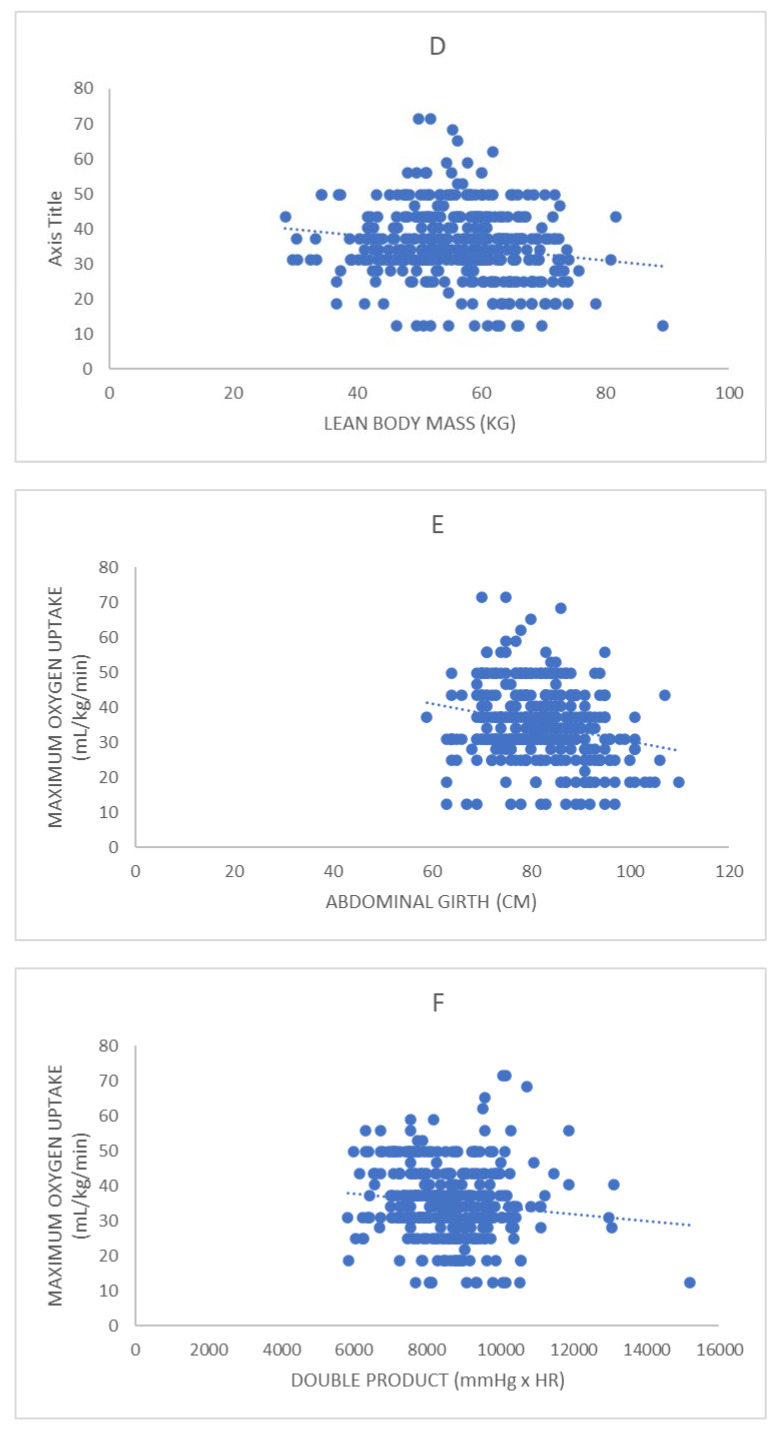
Correlation between the study participants’ maximum oxygen uptake and the anthropometric and health variables with which it maintained a significant correlation: (**A**) weight; (**B**) body mass index; (**C**) body fat percentage; (**D**) lean body mass; (**E**) abdominal girth; (**F**) double product.

As for the relationship between fitness test results and anthropometric and health variables (see Table 3 and Figure 1, Figure 2, Figure 3 and Figure 4), S&R presented a weak significant positive correlation with AG. SLJ presented a weak significant positive correlation with HE; weak significant negative with WE, LBM, WHR, SBP, DBP, A-BP, DP, GLU; and significant moderate negative with BMI, FAT, and AG. SHR presented a weak significant positive correlation with WE, BMI, AG, SBP, DP, and GLU; moderate significant positive with FAT; and weak significant negative with HE. Finally, VO2max presented a weak significant negative correlation with WE, BMI, FAT, LBM, AG, and DP.

Fitness test results maintained significant correlations with each other. S&R presented a weak significant positive correlation with SLJ, and VO2max. SLJ maintained a weak significant positive correlation with S&R; moderate significant positive with VO2max; and weak significant negative with SHR. SHR presented a weak significant negative correlation with SLJ and VO2max. Finally, VO2max presented a weak significant positive correlation with S&R, a moderate significant positive with SLJ, and a weak significant negative correlation with SHR.

The anthropometric variables analyzed in the present study (WE, HE, BMI, FAT, LBM, and WHR) also presented numerous significant correlations with each other, ranging the correlation coefficients from weak to very strong. Thus, AG and LBM correlated significantly with all the remaining anthropometric variables. WE had a significant correlation with all remaining anthropometric variables except WHR. Additionally, BMI and FAT significantly correlated with all remaining anthropometric variables except WHR and HE.

Similarly, the health variables analyzed (SBP, DBP, SPO2, A-BP, DP, and GLU) also presented significant correlations between themselves in some cases (see Table 2). In this regard, it should be noted that SBP significantly correlated with the rest of the health variables except SPO2.

Finally, certain anthropometric variables examined also maintained significant correlations with the selected health variables. Among them, WE, BM, LBM, and AG maintained significant correlations with all health variables except SPO2, whereas FAT significantly correlated with all health variables except SPO2 and DBP. In all previous cases, the correlation coefficients were weak or moderate.

Furthermore, as shown in Table 4, the multiple regression analysis conducted originated four models: Model 1 for S&R, model 2 for SLJ, model 3 for SHR, and model 4 for VO2max. The values obtained are shown in Table 3. According to the results of the mentioned analysis, it was observed that AG is a predictor variable of S&R. AG and—to a lesser extent—HE are predictors of SLJ. FAT, HE, and WE are—in decreasing order—predictor variables of SHR. Finally, AG is a predictor variable of VO2max. Additionally, the R^2^ values obtained (after performing the multiple regression analysis) were not higher than the Durbin-Watson values, and the Durbin-Watson scores were not close to 0. Thus, the possible existence of spurious relationships between variables was ruled out.

As for the logistic regression analysis (see Table 5), it was verified that SLJ and SHR results can predict BMI, FAT, and GLU, but with modest Nagelkerke R^2^ values in all three cases. Likewise, SLJ results and VO2max can predict AG and DBP. However, the Nagelkerke R^2^ values obtained were modest in both cases. Similarly, it was ascertained that SHR results can predict WHR and SBP variables. Nevertheless, in both cases, the Nagelkerke R^2^ values observed were poor. Finally, none of the fitness test results was useful to predict SPO2.

## 4. Discussion

The present study aimed to analyze the association between specific field-based physical fitness test results and selected health biomarkers variables to determine whether physical fitness can be a valid health marker. Once the results were analyzed, it was found that the marks obtained in the S&R test were only associated with AG. The multiple regression analysis confirmed this relationship, whereas the logistic regression showed that S&R does not have a predictive capacity for the selected anthropometric and health variables. There is a certain degree of agreement between these results and those obtained by Suni et al. (1998) [17] with adults and by the Institute of Medicine (2012) [39] with young people since, in both cases, no relationship was found between flexibility tests results and health markers. However, it should also be taken into account that very few studies have analyzed the association between flexibility and health markers in young people [39,40]. Therefore, more studies are warranted to verify possible correlations. Additionally, it should also be noted that flexibility is joint-specific. Thus, one person may have good levels of flexibility in the glenohumeral joint but not in the acetabulofemoral joint [39]. Likewise, since of all the anthropometric and health variables analyzed, only S&R correlated with AG—and despite some studies have verified that subjects with a higher percentage of fatty tissue present lower levels of flexibility [41,42]—it cannot be ruled out that this result is due to the presence of central adiposity, which may limit trunk flexion, and therefore, S&R results [43,44].

Moreover, S&R maintained a significant but weak correlation with SLJ and VO2max. In agreement with these results, Rahim et al. (2020) [45] also found a significant but weak correlation between jumping performance and S&R test marks in college athletes, and Briceño Torres and Moncada Jiménez [46] found a significant correlation between S&R marks and maximum oxygen uptake in middle-aged adults. In this regard, SLJ performance may be favored by greater hamstring flexibility as it may allow subjects to obtain greater forward momentum, as suggested in previous studies [45,47]. In addition, optimal levels of flexibility may contribute to endurance performance through improved running economy [48]. In contrast, Bogalho et al. (2022) and Siahkouhian et al. (2009) [49,50] found no relationship between jumping ability and S&R test scores in soccer players. Therefore, based on the results of the present study, S&R does not seem to be a good predictor of the selected health variables. As for its predictive capacity for the health- and skill-related fitness components, by virtue of the weak correlations found and the contradictory results of other studies, further research is needed to clarify this aspect.

Regarding the SLJ, significant (but weak or moderate) correlations were found between the results of this test and anthropometric parameters such as WE, LBM, WHR, BMI, FAT, and AG (which was also a predictor factor). This implies that higher levels of adiposity and higher BM and BMI result in worse scores and suggests that relative strength—in addition to power and jumping technique [51]—may determine SLJ performance, as demonstrated in previous studies [52,53]. SLJ also maintained a significant but weak correlation with HE (which was also a predictor factor). This result aligns with the findings of Giuriato et al. (2021) [54] since they verified that the greater HE and the lower WHR, the better the results obtained in SLJ by adolescents. Moreover, Singh Sidhu (2018) [51] points out that although HE conditions SLJ marks, further aspects also determine SLJ results, such as muscular strength and power, jumping technique, lower extremity length, BMI, and biomechanical factors [44]. For this reason, it is conceivable that in some studies (including the present research), only weak correlations between HE and SLJ are found.

The correlation between SLJ and the health parameters analyzed was also significant but weak for all the health parameters (SBP, DBP, A-BP, DP, GLU) except SPO2. These results cannot be compared with other studies because, to our knowledge, the relationship between the health variables included in this research and SLJ test scores has not been analyzed. Hence, new studies are needed to confirm the weak correlations found. In contrast, scientific evidence indicates that better SLJ performance is associated with improved bone density and body composition in youth [39]. Moreover, the significant but weak or moderate correlation found in this study between SLJ and the rest of the fitness tests conducted contrasts to some extent with the results obtained by Sánchez Medina et al. (2015) [55] with adolescents and by Prieto-González et al. (2021) [56] with college-age subjects, since in these two studies strong or very strong correlations were found. This discrepancy could be explained by the smaller and non-stratified sample size used in both studies. Nevertheless, a common aspect between the two studies above and the present research is that significant correlations were found between different fitness test scores. This finding reveals an interrelation between fitness components (i.e., power, agility, flexibility, cardiovascular endurance), although those components are different or opposed in nature.

SHR test results maintained a significant but weak or moderate correlation with all the anthropometric variables analyzed except WHR and LBM. Likewise, the multiple regression analysis revealed that FAT, HE, and WE are predictor variables of SHR scores, and the logistic regression showed that SHR can predict BMI, FAT, WHR, SBP, and GLU. These results confirm the findings of previous studies [54,57] and suggest that longer lower extremities length, greater relative strength, and lower adiposity levels influence agility performance, as verified in previous studies [58]. SHR significantly but weakly correlated with all the health variables analyzed except SPO2. This suggests that the predictive validity of SHR is limited for the selected variables. Previous studies reported positive correlations between agility performance and skeletal health [15]. However, few studies have examined the relationship between agility tests and health indicators [40]. Therefore, new studies examining the health-related predictive validity of SHR are needed. Finally, regarding the significant but weak correlation observed between SHR and SLJ and VO2max, it could be—as previously mentioned—due to the existence of interrelationship among different fitness components in non-athletic populations, although the fitness components required to perform in SHR successfully (speed and agility) and BT (cardiovascular endurance) are of opposite nature.

VO2max maintained a significant but weak correlation with most of the anthropometric variables examined (WE, BMI, FAT, LBM, AG). Although a higher level of correlation could be expected, these results coincide to some extent with previous studies in which an association between VO2max and BM and body composition were also observed [15,39]. Thus, it may be interpreted that lower body weight and adiposity improve running economy and therefore, BT performance [59]. Surprisingly, of all the health variables analyzed, VO2max only correlated significantly (but weakly) with DP. These results contrast with those reported by the Institute of Medicine (2012) [39]. They observed a clear association between cardiovascular performance and blood pressure. Hence, further studies might be conducted to clarify this discrepancy.

Regarding the anthropometric parameters and the relationships among themselves, Marques et al. (2021) [40] point out that there is no single measure to assess all body composition components in young people [60,61]. They add that BMI does not differentiate between fat and lean mass. Therefore, it is not a sufficient indicator of fat mass or abdominal adiposity. Thus, experts propose the use of AG to assess central adiposity. However, in the present study, BMI maintained a significantly strong correlation with FAT and a significant and very strong correlation with LBM and AG. Likewise, AG maintained a significantly strong correlation with FAT and LBM. These results coincide with the findings of Santos and Mota (2011) [8]. They verified that BMI, skinfolds, and waist circumference are valid and reliable measures of body fat. In the same vein, Ruiz et al. (2011) point out that skinfolds and BMI are valid tests for assessing body composition and waist circumference to estimate central fat [10].

Regarding the relationship between anthropometric and health parameters, previous studies reported a clear relationship between body composition and the risk of developing cardiovascular diseases and type II diabetes [62,63]. In contrast, in the present study, although BMI, FAT, LBM, and AG were significantly correlated with all the health parameters analyzed except SPO2, these correlations were weak or moderate in the case of BMI, FAT, and AG, and weak in the case of LBM.

Based on the above, analyzing the overall study results, it can be observed that performing properly in the fitness tests included in this research is necessary, though not sufficient to be healthy. Therefore, fitness tests are valid to measure physical condition but not entirely valid to measure the overall health status, only specific markers. Apart from physical fitness, other aspects might significantly condition health status, such as behavioral (nutritional, lifestyle, stress), social (education), and environmental factors (pollution, solar radiation, temperature and humidity, noise, contamination) [64]. Moreover, the global outcomes of the present research do not strongly concur with the claims of Santos et al. (2011) [8]. They consider cardiorespiratory fitness, muscle strength, and body composition (but not flexibility) valid health markers in adolescents. In the same vein, Ortega et al. (2008) [15] argue that physical fitness is associated with numerous health indicators, and the Institute of Medicine (2012) [39] points out that fitness tests are valid, simple, accurate, and low-cost tools for health monitoring. For this reason, several authors propose implementing public health policies and promoting physical activity and physical fitness assessment to reduce the incidence of communicable diseases [7,12]. In this context, schools might play a leading role [12,65,66]. However, based on the weak or moderate correlations observed in this study between fitness scores and anthropometric and health variables, together with the poor or modest predictive ability of the fitness tests (see Table 5), it could not be stated that physical fitness is one of the most important markers of health in youth, and a predictor of morbidity/mortality in adults as Fonseca Del Pozo et al. (2017) indicate [66]. In this regard, Ruiz et al. (2009) [16] argue that physical fitness and health do not maintain a clear relationship, probably due to the great variability of measurement methods and instruments used [18].

Therefore, in addition to promoting physical activity and physical condition assessment, future studies should determine which physical fitness tests are the most valid for assessing health status. Likewise, Physical Education and health professionals should be provided with inexpensive, simple, and accurate field-based instruments that complement physical condition assessment to evaluate health variables that do not correlate with physical fitness, such as SPO2 [67].

Finally, the limitations of the present study should be mentioned. First, a relatively limited number of fitness tests (four) and health variables (six) were analyzed. Therefore, it cannot be ruled out that including a higher number of measurements would have provided additional findings. Furthermore, the study was conducted only with males aged between 17 and 21. Finally, it should be mentioned that the present study did not carry out an analysis specifically aimed at detecting potential nonlinear relationships between variables. Although most similar studies do not perform this type of analysis, Zvonar et al. (2019) observed nonlinear correlations between results obtained in physical fitness tests and anthropometric variables. Therefore, future studies should also explore this possibility [68].

## 5. Conclusions

In college-aged males, SLJ and SHR present a weak-to-moderate validity to predict the selected anthropometric markers and weak to predict all the health indicators included in this study except SPO2. VO2max has only weak validity to predict the selected anthropometric markers, whereas S&R is not a valid test to predict the selected health or anthropometric markers. Anthropometric measurements have weak validity in predicting the selected health markers except for SPO2. By contrast, BMI and AG are valid, simple, and economical measurements to assess body fat. Moreover, among the subjects included in this study, there is a positive interaction between the results obtained in the field-based fitness tests conducted, although these tests assess fitness components of different or even opposite nature.

## 6. Practical Applications

Considering the null, weak or moderate correlations observed between specific field-based tests and selected anthropometric and health variables, future studies should aim to determine which physical fitness tests are the best predictors of individuals’ health status. Furthermore, to perform more comprehensive health assessments, in addition to using fitness tests, physical education teachers and health professionals might use simple, inexpensive, and accurate measurement instruments to assess health variables that are not correlated with fitness.

## Figures and Tables

**Table 1:** Ranges established for each of the anthropometric or health variables included in the binary logistic regression analysis.

Variable	Healthy or Acceptable Values	Unhealthy or Increased Risk Values
Body mass index (Kg/m^2^)	18.5–24.9	<18.5 or ≥25
Body fat percentage (%)	≤25.3	>25.3
Abdominal girth (cm)	<94	≥94
Waist-to-hip Ratio	<90	≥90
Systolic blood pressure (mmHg)	<140	≥140
Diastolic blood pressure (mmHg)	<90	≥90
Oxygen saturation (%)	95–100	≤94
Fasting blood glucose (mg/dL)	70–100	<70 or >100

**Table 2:** Characteristics of the participants.

Variable	X̅	SD	95% Confidence Interval
Age (years)	18.55	1.60	18.89–19.21
Weight (kg)	70.26	14.04	68.86–71.66
Height (cm)	175.93	4.06	1.74–1.77
Body mass index (Kg/m^2^)	23.45	3.69	23.08–23.81
Body fat percentage (%)	18.93	7.28	18.20–19.65
Lean body mass (kg)	56.40	9.85	55.42–57.39
Abdominal girth (cm)	81.79	8.72	80.92–82.67
Waist-to-hip ratio (index)	0.84	0.05	0.84–0.085
Systolic blood pressure (mmHg)	128.50	12.96	127.20–129.79
Diastolic blood pressure (mmHg)	73.68	8.54	72.83–74.54
Oxygen saturation (%)	97.43	5.25	96.90–97.95
Average blood pressure (mmHg)	91.95	7.89	91.17–92.74
Double product (mmHg × HR)	8595.53	1213.93	8474.67–8716.38
Fasting blood glucose (mg/dL)	98.40	5.66	97.84-98.97
Sit-and-reach test (cm)	0.75	9.12	−0.15–1.66
Standing long jump test (cm)	177.5	25.18	175.07–180.08
Shuttle run test (sec)	19.35	1.73	19.18–19.53
Maximum oxygen uptake (mL/kg/min)	35.10	16.60	32.07–38.09

**Table 3:** Correlation matrix: correlation between variables.

		WE	HE	BMI	FAT	LBM	AG	WHR	SBP	DBP	SPO2	A-BP	DP	GLU	S&R	SLJ	SHR	VO2max
AGE	*r*	0.06	0.07	0.05	−0.08	0.14 *	0.05	0.13 *	0.04	0.17 *	−0.01	0.16 *	−0.03	−0.04	−0.007	−0.01	−0.09	−0.03
	*p*	0.23	0.12	0.33	0.09	0.006	0.29	0.006	0.40	<0.001	0.77	0.003	0.52	0.39	0.88	0.79	0.052	0.79
WE	*r*		0.35 *	0.95 *	0.49 *	0.94 *	0.75 *	0.09	0.27 *	0.21 *	−0.01	0.29 *	0.34 *	0.34 *	−0.06	−0.24 *	0.18 *	−0.17 *
	*p*		<0.001	<0.001	<0.001	<0.001	<0.001	0.80	<0.001	<0.001	0.99	<0.001	<0.001	<0.001	0.21	<0.001	<0.001	0.001
HE	*r*			0.01	−0.9	0.44 *	0.13 *	0.06	0.06	0.11 *	0.11 *	0.11 *	0.05	0.06	−0.02	0.17 *	−0.20 *	−0.05
	*p*			0.77	0.06	<0.001	0.1	0.21	0.26	0.27	0.03	0.03	0.34	0.25	0.69	0.001	<0.001	0.22
BMI	*r*				0.62 *	0.73 *	0.83 *	0.09	0.33 *	0.22 *	−0.05	0.34 *	0.41 *	0.44 *	−0.05	−0.41 *	0.26 *	−0.17 *
	*p*				<0.001	<0.001	<0.001	0.07	<0.001	<0.001	0.28	<0.001	<0.001	<0.001	0.27	<0.001	<0.001	<0.001
FAT	*r*					0.26 *	0.57 *	0.07	0.16 *	0.03	0.02	0.11 *	0.27 *	0.43 *	−0.01	−0.31 *	0.30 *	−0.13 *
	*p*					<0.001	<0.001	0.13	0.001	0.56	0.71	0.03	<0.001	<0.001	0.77	<0.001	<0.001	0.01
LBM	*r*						0.63 *	−0.14 *	0.23 *	0.23 *	0.007	0.29 *	0.28 *	0.23 *	−0.06	−0.16 *	0.10	−0.17 *
	*p*						<0.001	<0.001	<0.001	<0.001	0.89	<0.001	<0.001	<0.001	0.28	0.001	0.06	0.001
AG	*r*							0.37 *	0.28 *	0.21 *	−0.05	0.30 *	0.35 *	0.38 *	0.13 *	−0.44 *	0.20 *	−0.23 *
	*p*							<0.001	<0.001	<0.001	0.31	<0.001	<0.001	<0.001	0.014	<0.001	<0.001	<0.001
WHR	*r*								0.13 *	0.041	−0.06	0.10	0.11 *	0.08	0.001	−0.20 *	−0.05	−0.008
	*p*								0.013	0.41	0.20	0.05	0.034	0.11	0.99	<0.001	0.35	0.88
SBP	*r*									0.23 *	−0.02	0.72 *	0.79 *	0.16 *	−0.06	−0.11 *	0.11 *	−0.05
	*p*									<0.001	0.65	<0.001	<0.001	0.002	0.21	0.02	0.03	0.33
DBP	*r*										−0.04	0.85 *	0.17 *	−0.08	−0.08	−0.12 *	0.006	−0.05
	*p*										0.43	<0.001	0.001	0.14	0.13	0.023	0.89	0.28
SPO2	*r*											−0.04	−0.01	−0.05	0.008	0.08	0.002	0.05
	*p*											0.42	0.79	0.30	0.87	0.14	0.96	0.33
A-BP	*r*												0.56 *	−0.03	−0.09	−0.15 *	0.07	0.09
	*p*												<0.001	0.50	0.08	0.004	0.19	0.08
DP	*r*													0.20 *	−0.03	−0.16 *	0.16 *	−0.20 *
	*p*													<0.001	0.50	0.001	0.002	<0.001
GLU	*r*														0.02	−0.19 *	0.19 *	−0.10
	*p*														0.73	<0.001	<0.001	0.06
S&R	*r*															0.24 *	−0.09	0.20 *
	*p*															<0.001	0.07	<0.001
SLJ	*r*																−0.21 *	0.30 *
	*p*																<0.001	<0.001
SHR	*r*																	−0.19 *
	*p*																	<0.001

Legend: *r*: Pearson correlation coefficient; *p*: *p*-value (*p* < 0.05); *: Significant correlation found; WE: weight; HE: height; BMI: body mass index; FAT: body fat percentage; LBM: lean body mass; AG: abdominal Girth; WHR: waist-to-hip ratio; SBP: systolic blood pressure, DBP: diastolic blood pressure; SPO2: Oxygen saturation; A-BP: average blood pressure; DP: double product; GLU: fasting blood glucose; S&R: sit-and-reach test; SLJ: standing long jump test; SHR: shuttle run test; VO2max: maximum oxygen uptake.

**Table 4:** Multiple linear regression analysis (using the stepwise method) of the relationship between fitness test results and AG, HE, FAT and WE.

Analysis	Dependent Variable	Included Independent Variable/s	R^2^	*p*-Value	Standardized Coecient (β)	*p*-Value
Model 1	S&R	AG	0.015	0.014	−0.12	0.009
Model 2	SLJ	AG HE	0.24	<0.001	−0.47 0.23	<0.001 <0.001
Model 3	SHR	FAT HE WE	0.13	<0.001	0.24 −0.19 0.17	<0.001 0.001 0.005
Model 4	VO2max	AG	0.05	<0.001	−0.23	<0.001

Legend: AG: abdominal girth; HE: height; FAT: body fat percentage; WE: weight; S&R: sit-and-reach test; SLJ: standing long jump test; SHR: shuttle run test; VO2max: maximum oxygen uptake.

**Table 5:** Logistic regression analysis to verify the fitness test results’ predictive ability for the following variables: BMI, FAT, AG, WHR, SBP, DBP, SPO2, and GLU.

D.V.	P.V.	Nagelkerke R-Square	B	S.E.	Wald	df	Sig	Exp(B)	95% CI for Exp(B)	
									Lower	Upper
BMI	S&R	0.152	0.023	0.012	3.58	1	0.058	1.024	0.999	1.05
	SLJ		−0.016	0.005	11.62	1	0.001 *	1.017	1.01	1.02
	SHR		−0.242	0.069	12.22	1	0.001 *	0.785	0.686	0.899
	VO2max		0.055	0.071	0.59	1	0.443	1.056	0.919	1.21
FAT	S&R	0.175	−0.010	0.015	0.414	1	0.520	0.990	0.961	1.021
	SLJ		−0.237	0.006	17.03	1	0.001 *	1.026	1.013	1.038
	SHR		0.026	0.079	8.91	1	0.003 *	0.789	0.675	0.922
	VO2max		0.104	0.089	1.37	1	0.241	1.109	0.933	1.320
AG	S&R	0.227	0.017	0.023	0.595	1	0.441	1.018	0.973	1.064
	SLJ		0.037	0.009	16.361	1	0.000 *	1.038	1.019	1.057
	SHR		−0.044	0.107	0.171	1	0.679	0.957	0.776	1.179
	VO2max		0.397	0.132	8.973	1	0.003 *	1.487	1.147	1.927
WHR	S&R	0.034	−0.012	0.014	0.637	1	0.425	0.989	0.961	1.017
	SLJ		0.004	0.006	0.392	1	0.531	1.004	0.992	1.015
	SHR		0.220	0.084	6.877	1	0.009 *	1.246	1.057	1.469
	VO2max		0.014	0.086	0.027	1	0.870	1.014	0.857	1.200
SBP	S&R	0.020	0.013	0.016	0.697	1	0.404	1.013	0.982	1.045
	SLJ		−0.001	0.006	0.035	1	0.851	0.999	0.988	1.010
	SHR		−0.155	0.078	3.945	1	0.047 *	0.857	0.735	0.998
	VO2max		−0.027	0.086	0.096	1	0.757	0.974	0.822	1.153
DBP	S&R	0.023	0.017	0.023	0.595	1	0.441	1.018	0.973	1.064
	SLJ		0.037	0.009	16.361	1	0.000 *	1.038	1.019	1.057
	SHR		−0.044	0.107	0.171	1	0.679	0.957	0.776	1.179
	VO2max		0.397	0.132	8.973	1	0.003 *	1.487	1.147	1.927
SPO2	S&R	0.001	−0.006	0.026	0.049	1	0.825	0.994	0.944	1.047
	SLJ		0.016	0.011	2.161	1	0.142	1.016	0.995	1.037
	SHR		0.112	0.146	0.593	1	0.441	1.119	0.841	1.489
	VO2max		0.094	0.158	0.351	1	0.554	1.098	0.806	1.497
GLU	S&R	0.117	−0.009	0.012	0.576	1	0.448	0.991	0.968	1.015
	SLJ		0.020	0.005	16.854	1	0.000 *	1.020	1.010	1.030
	SHR		−0.202	0.066	9.260	1	0.002 *	0.817	0.718	0.931
	VO2max		−0.007	0.069	0.009	1	0.923	0.993	0.868	1.137

Legend: D.V.: dependent variable; P.V.: predictor variable; B: beta value; S.E.: standard error; d.f.: degrees of freedom; Sig: statistical significance level; *: indicates that the variable has a statistically significant predictive ability in the model; Exp(B): exponential value of B; BMI: body mass index; FAT: body fat percentage; AG: abdominal Girth; WHR: waist-to-hip ratio; SBP: systolic blood pressure, DBP: diastolic blood pressure; SPO2: oxygen saturation; GLU: fasting blood glucose; S&R: sit-and-reach test; SLJ: standing long jump test; SHR: shuttle run test; VO2max: maximum oxygen uptake.

## Data Availability

Not applicable.

## References

[1] World Health Organization The Global Health Observatory. https://www.who.int/data/gho/data/major-themes/health-and-well-being#:~:text=The%20WHO%20constitution%20states%3A%20%22Health,of%20mental%20disorders%20or%20disabilities.

[2] Brooks J., Ahmad I., Easton G. (2016). Promoting physical activity: The general practice agenda. Br. J. Gen. Pract. J. R. Coll. Gen. Pract..

[3] Polet J., Hassandra M., Lintunen T., Laukkanen A., Hankonen N., Hirvensalo M., Tammelin T., Hagger M.S. (2019). Using physical education to promote out-of school physical activity in lower secondary school students—A randomized controlled trial protocol. BMC Public Health.

[4] Sylvia L.G., Bernstein E.E., Hubbard J.L., Keating L., Anderson E.J. (2014). Practical guide to measuring physical activity. J. Acad. Nutr. Diet..

[5] Macfarlane D.J., Lee C.C., Ho E.Y., Chan K.L., Chan D. (2006). Convergent validity of six methods to assess physical activity in daily life. J. Appl. Physiol..

[6] Reilly J.J., Penpraze V., Hislop J., Davies G., Grant S., Paton J.Y. (2008). Objective measurement of physical activity and sedentary behaviour: Review with new data. Arch. Dis. Child..

[7] Grant C.C., Janse van Rensburg D., Pepper M., du Toit P.S., Wood P., Ker J., Krüger P., Grobbelaar C.J., Nolte K., Fletcher F. (2014). The correlation between the health-related fitness of healthy participants measured at home as opposed to fitness measured by sport scientists in a laboratory. S. Afr. Fam. Pract..

[8] Santos R., Mota J. (2011). The ALPHA health-related physical fitness test battery for children and adolescents. Nutr. Hosp..

[9] Raghuveer G., Hartz J., Lubans D.R., Takken T., Wiltz J.L., Mietus-Snyder M., Perak A.M., Baker-Smith C., Pietris N., Edwards N.M. (2020). Cardiorespiratory Fitness in Youth: An Important Marker of Health: A Scientific Statement from the American Heart Association. Circulation.

[10] Ruiz J.R., España Romero V., Castro Piñero J., Artero E.G., Ortega F.B., Cuenca García M., Jiménez Pavón D., Chillón P., Girela Rejón M.J., Mora J. (2011). ALPHA-fitness test battery: Health-related field-based fitness tests assessment in children and adolescents. Nutr. Hosp..

[11] National Curriculum in England: PE Programmes of Study. https://www.gov.uk/government/publications/national-curriculum-in-england-physical-education-programmes-of-study.

[12] Real Decreto 1105/2014, de 26 de Diciembre, por el que se Establece el Currículo Básico de la Educación Secundaria Obligatoria y del Bachillerato [Royal Decree 1105/2014, of December 26, Establishing the Basic Curriculum for Compulsory Secondary Education and the Baccalaureate]. https://www.boe.es/buscar/act.php?id=BOE-A-2015-37.

[13] Etches V., Frank J., Di Ruggiero E., Manuel D. (2006). Measuring population health: A review of indicators. Annu. Rev. Public Health.

[14] Zeiher J., Ombrellaro K.J., Perumal N., Keil T., Mensink G., Finger J.D. (2019). Correlates and Determinants of Cardiorespiratory Fitness in Adults: A Systematic Review. Sport. Med.-Open.

[15] Ortega F.B., Ruiz J.R., Castillo M.J., Sjöström M. (2008). Physical fitness in childhood and adolescence: A powerful marker of health. Int. J. Obes..

[16] Ruiz J.R., Castro-Piñero J., Artero E.G., Ortega F.B., Sjöström M., Suni J., Castillo M.J. (2009). Predictive validity of health-related fitness in youth: A systematic review. Br. J. Sport. Med..

[17] Suni J.H., Oja P., Miilunpalo S.I., Pasanen M.E., Vuori I.M., Bös K. (1998). Health-related fitness test battery for adults: Associations with perceived health, mobility, and back function and symptoms. Arch. Phys. Med. Rehabil..

[18] Martínez-Vizcaíno V., Sánchez-López M. (2008). Relación entre actividad física y condición física en niños y adolescentes [Relationship between physical activity and physical fitness in children and adolescents]. Rev. Esp. Cardiol..

[19] Bryce-Moncloa A., Alegría-Valdivia E., San Martin-San Martin M.G. (2017). Cardiovascular risk and obesity. An. Fac. Med..

[20] Kothari C.R. (2009). Research Methodology: Methods and Techniques.

[21] González-Gallego J., Collado P.S., Mataix J. (2006). Nutrición en el deporte. Ayudas Ergogénicas y Dopaje. [Nutrition in sport. Ergogenic aids and doping].

[22] Eguchi K., Kuruvilla S., Ogedegbe G., Gerin W., Schwartz J.E., Pickering T.G. (2009). What is the optimal interval between successive home blood pressure readings using an automated oscillometric device?. J. Hypertens..

[23] Pocock G., Richards C.D. (2009). The Human Body: An Introduction for the Biomedical and Health Sciences.

[24] Mayorga-Vega D., Merino-Marban R., Viciana J. (2014). Criterion-Related Validity of Sit-and-Reach Tests for Estimating Hamstring and Lumbar Extensibility: A Meta-Analysis. J. Sport. Sci. Med..

[25] López-Miñarro P.A., Muyor J.M., Belmonte F., Alacid F. (2012). Acute effects of hamstring stretching on sagittal spinal curvatures and pelvic tilt. J. Hum. Kinet..

[26] Reid C., Dolan M., De Beliso M. (2017). The Reliability of the Standing Long Jump in NCAA Track and Field Athletes. Int. J. Sport. Sci..

[27] Martínez López E.J. (2004). Application of the speed test 10 × 5 meters, sprint of 20 meters and tapping-test with the arms. Results and statistic analysis in Secondary Education. Rev. Int. Med. Cienc. Act. Física Deporte.

[28] Christiansen C., Petersen M., Froberg K., Hansen L., Wedderkopp N. The 10 × 5 metre shuttle run test may be a good predictor of aerobic performance in pre-pubertal children. Proceedings of the Nordic Conference 2010. Interdisciplinary Perspectives on Health, Participation and Effects of Sport and Exercise 2010.

[29] Gastón C.G., Secchib J.D. (2014). 20 meters shuttle run test with stages of one minute. An original idea that has lasted for 30 years. Apunts.

[30] Paradisis G.P., Zacharogiannis E., Mandila D., Smirtiotou A., Argeitaki P., Cooke C.B. (2014). Multi-Stage 20-m Shuttle Run Fitness Test, Maximal Oxygen Uptake and Velocity at Maximal Oxygen Uptake. J. Hum. Kinet..

[31] Hazra A., Gogtay N. (2016). Biostatistics Series Module 6: Correlation and Linear Regression. Indian J. Dermatol..

[32] World Health Organization Obesity: Preventing and Managing the Global Epidemic: Report of a WHO Consultation. https://apps.who.int/iris/handle/10665/42330.

[33] Branco B., Bernuci M.P., Marques D.C., Carvalho I.Z., Barrero C., de Oliveira F.M., Ladeia G.F., Júnior N.N. (2018). Proposal of a normative table for body fat percentages of Brazilian young adults through bioimpedanciometry. J. Exerc. Rehabil..

[34] World Health Organization Waist Circumference and Waist-Hip Ratio: Report of a WHO Expert Consultation. https://www.who.int/publications/i/item/9789241501491.

[35] World Health Organization Noncommunicable Diseases: Hypertension. https://www.who.int/news-room/questions-and-answers/item/noncommunicable-diseases-hypertension.

[36] World Health Organization Oxygen. https://www.who.int/news-room/questions-and-answers/item/oxygen#:~:text=The%20normal%20range%20of%20SpO2,of%20altitude%20.

[37] World Health Organization Mean Fasting Blood Glucose. https://www.who.int/data/gho/indicator-metadata-registry/imr-details/2380#:~:text=The%20expected%20values%20for%20normal,and%20monitoring%20glycemia%20are%20recommended.

[38] Muijs D. (2011). Doing Quantitative Research in Education with SPSS.

[39] Institute of Medicine (2012). Fitness Measures and Health Outcomes in Youth.

[40] Marques A., Henriques-Neto D., Peralta M., Martins J., Gomes F., Popovic S., Masanovic B., Demetriou Y., Schlund A., Ihle A. (2021). Field-Based Health-Related Physical Fitness Tests in Children and Adolescents: A Systematic Review. Front. Pediatr..

[41] Bittencourt A., Vieira P., Ferreira M., Primo L., Deiró T., Avelino P., Menezes K., Lage S., Costa H. (2017). The Impact of Overweight on Flexibility and Functional Capacity. J. Nov. Physiother..

[42] Bataweel E.A., Ibrahim A.I. (2020). Balance and musculoskeletal flexibility in children with obesity: A cross-sectional study. Ann. Saudi Med..

[43] Lee P.F., Ho C.C., Kan N.W., Yeh D.P., Chang Y.C., Li Y.J., Tseng C.Y., Hsieh X.Y., Chiu C.H. (2020). The Association between Physical Fitness Performance and Abdominal Obesity Risk among Taiwanese Adults: A Cross-Sectional Study. Int. J. Environ. Res. Public Health.

[44] Chen H.H., Chen H.L., Lin Y.T., Lin C.W., Ho C.C., Lin H.Y., Lee P.F. (2020). The Associations between Functional Fitness Test Performance and Abdominal Obesity in Healthy Elderly People: Results from the National Physical Fitness Examination Survey in Taiwan. Int. J. Environ. Res. Public Health.

[45] Rahim M.A., Lee E.L., Malek N.J., Nadzalan D.S. (2020). Relationship Between Physical Fitness and Long Jump Performance. Int. J. Sci. Technol. Res..

[46] Briceño Torres J.M., Moncada Jiménez J. (2016). Physical health and stress in security officers of the University of Costa Rica in 2014. Rev. Costarric. Salud Pública.

[47] Church J.B., Wiggins M.S., Moode F.M., Crist R. (2001). Effect of warm up and flexibility treatments on vertical jump performance. J. Strength Cond. Res..

[48] Konrad A., Močnik R., Nakamura M., Sudi K., Tilp M. (2021). The Impact of a Single Stretching Session on Running Performance and Running Economy: A Scoping Review. Front. Physiol..

[49] Bogalho D., Gomes R., Mendes R., Dias G., Castro M.A. (2022). Impact of Flexibility on Vertical Jump, Balance and Speed in Amateur Football Players. Appl. Sci..

[50] Siahkouhian M., Faramoushi M., Hedayatnejad M. (2009). Correlation of vertical jump performance with flexibility in young soccer players. Med. Dello Sport.

[51] Singh Sidhu J. (2018). Physical Attributes as Indicator of Performance for Broad Jumping. Int. J. Curr. Res. Rev..

[52] Comfort P., Stewart A., Bloom L., Clarkson B. (2014). Relationships between strength, sprint, and jump performance in well-trained youth soccer players. J. Strength Cond. Res..

[53] Soto-García D., Díaz-Cruz J., Bautista I.J., Martínez Martín I. (2022). Effects of a Strength Training Protocol with Self-loading and Plyometry on Handball Physical Performance: First National Female Category. Cienc. Deporte.

[54] Giuriato M., Kawczynski A., Mroczek D., Lovecchio N., Nevill A. (2021). Allometric association between physical fitness test results, body size/shape, biological maturity, and time spent playing sports in adolescents. PLoS ONE.

[55] Sánchez Medina L., Domínguez Montes J.A., González Badillo J.J., Rodríguez Rosell D. (2015). Anthropometrics variables and performance in children of 10–15 years old. RETOS-Nuevas Tend. Educ. Física Deporte Recreación.

[56] Prieto-González P., Sagat P., Sedlacek J. (2021). Relationship between BMI and physical fitness in college-age males: A cross-sectional study. South Afr. J. Res. Sport Phys. Educ. Recreat..

[57] Carnevale Pellino V., Giuriato M., Ceccarelli G., Codella R., Vandoni M., Lovecchio N., Nevill A.M. (2020). Explosive Strength Modeling in Children: Trends According to Growth and Prediction Equation. Appl. Sci..

[58] Begu B., Kryeziu A.R., Bahtiri A. (2018). The influence of anthropometric variables in agility abilities of young basketball players. Sport Sci..

[59] Genton L., Mareschal J., Karsegard V.L., Achamrah N., Delsoglio M., Pichard C., Graf C., Herrmann F.R. (2019). An Increase in Fat Mass Index Predicts a Deterioration of Running Speed. Nutrients.

[60] Ross R., Neeland I.J., Yamashita S., Shai I., Seidell J., Magni P., Santos R.D., Arsenault B., Cuevas A., Hu F.B. (2020). Waist circumference as a vital sign in clinical practice: A Consensus Statement from the IAS and ICCR Working Group on Visceral Obesity. Nature reviews. Endocrinology.

[61] Xi B., Zong X., Kelishadi R., Litwin M., Hong Y.M., Poh B.K., Steffen L.M., Galcheva S.V., Herter-Aeberli I., Nawarycz T. (2020). International Waist Circumference Percentile Cutoffs for Central Obesity in Children and Adolescents Aged 6 to 18 Years. J. Clin. Endocrinol. Metab..

[62] Chen Y., He D., Yang T., Zhou H., Xiang S., Shen L., Wen J., Chen S., Peng S., Gan Y. (2020). Relationship between body composition indicators and risk of type 2 diabetes mellitus in Chinese adults. BMC Public Health.

[63] Liberato S.C., Maple-Brown L., Bressan J., Hills A.P. (2013). The relationships between body composition and cardiovascular risk factors in young Australian men. Nutr. J..

[64] Woolf S.H., Aron L., National Research Council (US), Institute of Medicine (US) (2013). Health in International Perspective: Shorter Lives, Poorer Health.

[65] Yang D.H., Zhu X., Haegele J.A., Wilson P.B.P., Wu X. (2019). The association between health-related fitness and physical activity during weekdays: Do fit students exercise more after school?. Sustainability.

[66] Fonseca Del Pozo F.J., Alonso J.V., Álvarez M.V., Orr S., Cantarero F. (2017). Physical fitness as an indicator of health status and its relationship to academic performance during the prepubertal period. Health Promot. Perspect..

[67] Lang J.J., Wolfe Phillips E., Orpana H.M., Tremblay M.S., Ross R., Ortega F.B., Silva D., Tomkinson G.R. (2018). Field-based measurement of cardiorespiratory fitness to evaluate physical activity interventions. Bull. World Health Organ..

[68] Zvonar M., Kasović M., Štefan L. (2019). Anthropometric Indices and Some Aspects of Physical Fitness in Croatian Adolescents by Gender. Int. J. Environ. Res. Public Health.

